# Oral Vitamin B12 Replacement for the Treatment of Pernicious Anemia

**DOI:** 10.3389/fmed.2016.00038

**Published:** 2016-08-23

**Authors:** Catherine Qiu Hua Chan, Lian Leng Low, Kheng Hock Lee

**Affiliations:** ^1^Department of Family Medicine and Continuing Care, Singapore General Hospital, Singapore; ^2^Family Medicine, Duke-NUS Medical School, Singapore

**Keywords:** oral vitamin B12, pernicious anemia, mecobalamin, cobalamin, cyanocobalamin

## Abstract

Many patients with pernicious anemia are treated with lifelong intramuscular (IM) vitamin B12 replacement. As early as the 1950s, there were studies suggesting that oral vitamin B12 replacement may provide adequate absorption. Nevertheless, oral vitamin B12 replacement in patients with pernicious anemia remains uncommon in clinical practice. The objective of this review is to provide an update on the effectiveness of oral vitamin B12 for the treatment of pernicious anemia, the recommended dosage, and the required frequency of laboratory test and clinical monitoring. Relevant articles were identified by PubMed search from January 1, 1980 to March 31, 2016 and through hand search of relevant reference articles. Two randomized controlled trials, three prospective papers, one systematic review, and three clinical reviews fulfilled our inclusion criteria. We found that oral vitamin B12 replacement at 1000 μg daily was adequate to replace vitamin B12 levels in patients with pernicious anemia. We conclude that oral vitamin B12 is an effective alternative to vitamin B12 IM injections. Patients should be offered this alternative after an informed discussion on the advantages and disadvantages of both treatment options.

## Introduction

Vitamin B12 deficiency is a common condition, and many are undiagnosed. Absolute deficiency occur up to 6% of those aged 60 years and older, whereas marginal deficiency occur in close to 20% of patients in later life (1). The manifestation of Vitamin B12 deficiency ranges from subtle, non-specific clinical features to serious neurological and neuropsychiatric complication if left untreated. With an aging population, screening for vitamin B12 level as part of anemia and cognitive impairment workup is more common. More cases are diagnosed, resulting in rising incidence of patients with vitamin B12 deficiency. The common causes of vitamin B deficiency are food-cobalamin (vitamin B12) malabsorption and pernicious anemia.

Pernicious anemia is an autoimmune gastritis resulting from the destruction of gastric parietal cells and consequent impairment of intrinsic factors secretion to bind the ingested vitamin B12. Other autoimmune disorders, especially thyroid disease, diabetes mellitus, and vitiligo, are also commonly associated with pernicious anemia. The cost and availability of auto-antibodies testing, such as intrinsic factor and anti-parietal cell antibodies, can be a barrier to further investigation for vitamin B12 deficiency to exclude pernicious anemia. Therefore, the exact prevalence of pernicious anemia is difficult to ascertain. It has been estimated that the prevalence of pernicious anemia in European countries is approximately 4% of the population (2). It is also well acknowledged that the prevalence increase with age and therefore more common in the elderly.

For patients with pernicious anemia, lifelong vitamin B12 therapy is indicated. Vitamin B12 is absorbed in the terminal ileum. This absorption is almost entirely dependent on intrinsic factor binding to vitamin B12. This bound complex in turn binds to the cubam receptor in the terminal ileum and is internalized. The complex is eventually released from lysosomes and transported across the cell membrane bound to transcobalamin in the blood circulation. Traditionally, vitamin B12 replacement is administered intramuscularly. However, it is believed that oral vitamin B12 can be absorbed passively independent of intrinsic factors. Passive diffusion accounts for about 1% of total absorption, and this route of absorption is unaffected in patients with pernicious anemia (3).

There were studies in the 1950s and the1960s that showed that oral vitamin B12 could be absorbed by patients with pernicious anemia and could lead to resolution of the anemia (3–6). However, lifelong intramuscular (IM) injection for replacement is still a common practice. In 1991, a survey done on Minneapolis internists led one commentary to conclude that oral vitamin B12 replacement for pernicious anemia was one of “medicine’s best kept secret” (7). In 1996, when the survey was repeated again in the same area, awareness and use of oral vitamin B12 for pernicious anemia had increased substantially (0–19%), but the majority of doctors still remain unaware of this treatment option (61%) (8).

The objective of our review is to inform clinicians on the effectiveness of oral vitamin B12 as adequate replacement in patients with pernicious anemia, as well as make recommendations on the dosing and frequency of clinical and laboratory monitoring.

## Methods

A PubMed search was conducted in April 2016 to identify suitable articles published from January 1, 1980 to March 31, 2016. The following search strategy was applied: “Administration, Oral” (MeSH term) AND “Vitamin B12” (MeSH term) AND “Anaemia, Pernicious” (MeSH term). Only studies evaluating the effectiveness of oral vitamin B12 replacement on pernicious anemia patients in entirety or as a subset were included for review. The search was limited to English articles.

The search strategy was summarized in the following flow chart.

### Flowchart on Selection of Articles


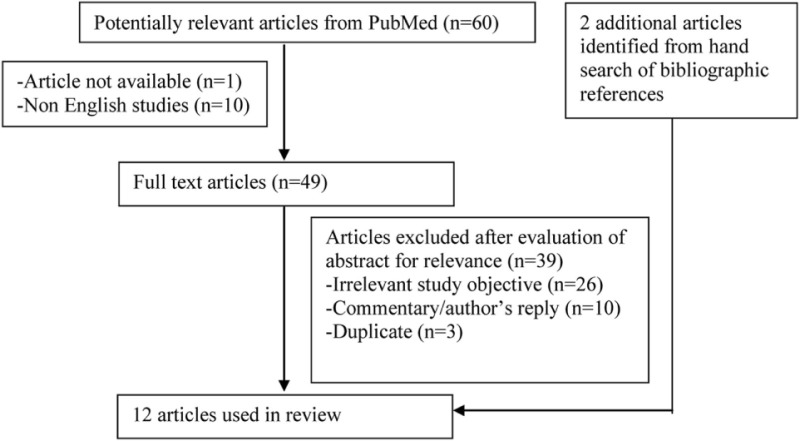


Grading of level of evidence and strength of recommendation was based on strength of recommendation taxonomy (SORT) framework (9). The description and level of evidence of included studies are shown in Table 1.

**Table 1:** **Description and level of evidence for articles reviewed**.

Study and study type	Participants, sample size, follow-up duration	Intervention, outcome measure	Results	Level of evidence (based on SORT)
Kuzminski et al. (10), randomized controlled trial (RCT), not blinded	Newly diagnosed vitamin B12-deficient patients	Oral vitamin B12 2000 μg daily for 120 days vs. intramuscular (IM) vitamin B12 1000 μg on days 1, 3, 7, 10, 14, 21, 30, 60, and 90	Serum vitamin B12 levels were significantly higher in the oral compared with IM group (643 ± 328 vs. 306 ± 118 pg/mL; *p* < 0.001) at 2 months. The difference was even greater at 4 months (1005 ± 595 vs. 325 ± 165 pg/mL)	2
	*N* (total number) = 33	Primary outcomes: serum vitamin B12, methylmalonic acid, homocysteine neurologic responses	Four of the 18 in the oral group and 4 of the 15 in the IM group had a neurological response with a marked improvement or clearing of paresthesia, ataxia, or memory loss	
	Intervention = 18 (5 had pernicious anemia)			
	Control = 15 (2 had pernicious anemia)			
	120 days			
Bolaman et al. (11), RCT, not blinded	Megaloblastic anemia due to vitamin B12 deficiency	Oral vitamin B12 1000 μg daily for 90 days vs. IM vitamin B12 1000 μg daily for 10 days, then once weekly for 28 days and after that continued with once monthly	Serum vitamin B12 levels increased in both groups at 90 days (oral group 213.8 pg/mL and IM group 225.5 pg/mL). There was a statistically significant difference between day 0 and day 90 in both groups (*p* < 0.0001) but authors did not analyze difference between both groups	2
	*N* = 60	Primary outcomes: serum vitamin B12, hemoglobin, platelet count, MCV, WBC, mini-mental state examination, neurological assessment	Both groups reported improvements of cognitive functions, sensory neuropathy, and vibration sense, but there was no statistical significant difference between both groups	
	Intervention = 26 (8 had presence of anti-parietal call antibody)			
	Control = 34 (3 had presence of anti-parietal call antibody)			
	90 days			
Delpre et al. (12), Prospective, open-label	Vitamin B12 deficiency	Sublingual vitamin B12 1000 μg daily for 7–12 days	Normalization of serum vitamin B12 levels was seen in all patients. An increase in vitamin B12 level was as much as fourfold compared with pretreatment in most patients. The mean change of 387.7 pg/mL was statistically significant (*p* = 0.0001, Student’s *t*-test)	3
	*N* = 18 (inclusive of patients with pernicious anemia but did not state number)	Primary outcome: serum vitamin B12		
	7–12 days			
Nyholm et al. (13), Prospective, case series	Vitamin B12 deficiency	Loading dose of IM vitamin B12 till vitamin B12 level reached lower 25th centile (418 pg/mL) and then converted to oral vitamin B12 1000 μg daily	Oral vitamin B12 was effective in all the patients (no patients had to restart IM vitamin B12). At 3 months, the median serum vitamin B12 level was 1193 pg/mL	3
	*N* = 40 (10 patients had pernicious anemia)	Primary outcomes: serum vitamin B12, hemoglobin, MCV, homocysteine, and neurological assessment	Oral treatment did not result in any new neurological complications	
	3–18 months			
Andres et al. (14), Prospective, open-label	Pernicious anemia	Oral vitamin B12 1000 μg daily for 3 months	After 3 months, serum vitamin B12 levels were increased in all 9 patients (mean increase, 117.4 pg/mL; *p* < 0.001). One patient’s result was not available due to technical problem	3
	*N* = 10	Primary outcome: serum vitamin B12, secondary outcomes: hemoglobin, platelet count, and MCV		
	3 months			

## Results

Sixty articles were identified through the electronic database search. Non-English studies were excluded. The abstracts of the remaining 49 articles were evaluated for relevance. Articles that did not involve or discuss about patients with pernicious anemia treated with oral vitamin B12 replacement were excluded. Duplicated articles and commentary/author’s reply were excluded. Another two articles were identified from hand search of bibliographic references of the shortlisted articles. A total of 12 articles [2 randomized controlled trials (RCTs) (10, 11), 3 prospective studies (12–14), 1 systematic review (15), and 6 clinical reviews (2, 16–20)] were obtained for the review.

### Effectiveness of Oral Vitamin B12

The data given in the studies all supported the use of oral vitamin B12 as a valid and effective way of treating vitamin B12 deficiency, including pernicious anemia. The age range of the study population was 23–92 years old. The RCT by Kuzminski et al. (10) and prospective study by Delpre et al. (12) were done in America. The RCT by Bolaman et al. (11) was done in Turkey. The other two prospective studies by Nyholm et al. (13) and Andres et al. (14) were done in United Kingdom and France, respectively. The studies were conducted during the period of late 1990s to early 2000s.

In Kuzminski’s study, serum vitamin B12 levels were significantly higher in the oral (vitamin B12 2000 μg) compared with IM (vitamin B12 1000 μg) group (643 ± 328 vs. 306 ± 118 pg/mL; *p* < 0.001) at 2 months. The difference was even greater at 4 months (1005 ± 595 vs. 325 ± 165 pg/mL). Five of the patients who had pernicious anemia in the oral vitamin B12 replacement group all had increase in serum vitamin B12 level. Four of the 18 in the oral group and 4 of the 15 in the IM group had a neurological response with a marked improvement or clearing of paresthesia, ataxia, or memory loss.

For Bolaman’s study, there was also an increase in serum vitamin B12 levels in both groups (oral vitamin B12 1000 μg vs. IM vitamin B12 1000 μg) at 90 days (Oral group 213.8 pg/mL and IM group 225.5 pg/mL). There was a statistically significant difference between days 0 and 90 in both groups (*p* < 0.0001), but authors did not analyze difference between both groups. Both groups reported improvements of cognitive functions, sensory neuropathy, and vibration sense, but there was no statistical significant difference between both groups.

The systematic review by Butler et al. (15) done on these two RCTs concluded that high oral doses of vitamin B12 could be as effective as IM administration in achieving short-term hematological and neurological responses. However, the two RCTs were limited by their small sample size and short follow-up period.

The rest of studies also had small sample size but some had longer follow-up period (up to 18 months). Normalization of serum vitamin B12 levels was seen in all patients (inclusive of patients with pernicious anemia) in Delpre’s study. An increase in vitamin B12 level was as much as fourfold compared with pretreatment in most patients. The mean change of 387.7 pg/mL was significant (*p* = 0.0001 in Student’s *t*-test). Oral vitamin B12 was effective in all the patients (10 patients had pernicious anemia) in Nyholm’s study with the median serum vitamin B12 level of 1193 pg/mL after 3 months of treatment. It was also reported that using oral treatment did not result in any new neurological complications. Andres’s study was the only study done on patients with pernicious anemia in entirety. All nine patients’ serum vitamin B12 levels improved (mean increase, 117.4 pg/mL; *p* < 0.001) after 3 months.

### Dosage of Oral Vitamin B12 Replacement Required

In all the five studies, an oral (Delpre’s study via sublingual route) dose of 1000 μg vitamin B12 was used with the exception of Kuzminski’s study, whereby a higher dose of 2000 μg was used. It had been showed that oral vitamin B12 at 1000 μg was adequate replacement in pernicious anemia patients.

There was a dose-finding trial done by Eussen et al. (21), and the results indicated that the lowest dose of oral vitamin B12 required to normalize biochemical markers of mild vitamin B12 deficiency in older people was more than 200 times greater than the recommended dietary allowance for vitamin B12 of approximately 3 μg/day. However, this study did not distinguish the extent to which differences in individual responses were due to active as opposed to passive absorption of vitamin B12.

In some of the clinical reviews, it was stated that many do not use oral vitamin B12 replacement in view of concern on the unpredictable absorption at low doses of oral replacement. Daily vitamin B12 turnover rate is about 2 μg/day, so an oral dose of 100–250 μg/day is sufficient for normal patients. However, in view of the estimated 1% of total absorption *via* passive diffusion in patients with pernicious anemia, a 1000 μg daily dose is recommended.

### Frequency of Monitoring

The outcome measurement of vitamin B12 level was done from a range of 1–3 months (exception of Delpre’s study, which was done ranging from 9 to 14 days). Improvement in vitamin B12 was seen as early as within a month. Only in Nyholm’s study, 11 of their patients were followed up until 18 months, whereby the vitamin B12 levels were maintained.

Close monitoring monthly is necessary at the start of oral replacement to verify normalization of lab results and monitoring for symptoms. Thereafter, annual monitoring should suffice. It should be carried out on a regular interval that is safe for patients and acceptable to both patients and doctors.

## Discussion

We summarized the level of evidence of using oral vitamin B12 replacement for patients with pernicious anemia (Table 1). The strength of clinical recommendations based on the SORT framework is provided in Table 2.

**Table 2:** **SORT recommendations for clinical practice**.

Clinical recommendation	Strength of recommendation
Oral vitamin B12 can be used for adequate replacement in patients with pernicious anemia	B
An oral vitamin B12 dose at 1000 μg is adequate replacement in patients with pernicious anemia	B
Close monitoring monthly is necessary at the start of oral replacement to verify normalization of lab results and monitoring for symptoms	C
Thereafter, annual monitoring should suffice	
Elevated serum homocysteine and methylmalonic acid levels should be included in future assessments of pernicious anemia and corrected to normal levels in patients with pernicious anemia	C

There is no “gold standard” in testing for vitamin B12 deficiency. It has been recommended that serum total homocysteine (Hcy) and methylmalonic acid (MMA) levels are more sensitive indicators of vitamin B12 status in pernicious anemia patients without any other disorders of vitamin B12 metabolism. In our review, some (10, 13), but not all, studies determine metabolite levels (Hcy and MMA) in pernicious anemia to assess effectiveness of therapy with vitamin B12. Most patients with pernicious anemia are not screened genetically for confirmatory causes of the disease. There are genetic errors of metabolism where serum vitamin B12 levels are normal, but there is a functional (cellular) level deficiency of the micronutrient that is often overlooked unless Hcy and MMA levels are also measured. Both Hcy and MMA levels are elevated in patients with vitamin B12 deficiency. Elevated serum Hcy and MMA levels (>3 SDs above the mean in normal subjects) have a sensitivity of 95.9 and 98.4%, respectively, to diagnose vitamin B12 deficiency (22). The levels decrease immediately after treatment and repeat measurements have clinical utility to document adequate vitamin B12 replacement. However, considerations of using Hcy and MMA levels would include cost, availability of the test, as well as having standardized reference intervals.

Patients with vitamin B12 deficiency who are symptomatic have severe neurological deficits or have critically low blood levels of vitamin B12 should be treated with IM administration. This is to ensure rapid replenishment of body stores to prevent irreversible consequences of deficiency. Subsequently, patients may be able to convert to oral replacement with close monitoring. For long-term maintenance therapy, oral vitamin B12 replacement can be effective in patients with pernicious anemia. Patient preference should be taken into consideration in the choice of treatment options. Few studies had included surveying patients regarding preference of choice between oral vs. IM replacement of vitamin B12. In Delpre’s study, 87% of them preferred tablets to injection (12). Eighty-seven percent found the tablets highly acceptable, while the remaining 13% agreed that tablets were acceptable. It must also be considered that adherence is likely to be better if the patient’s preferred route of administration is taken into consideration.

Additional factors to consider when helping patient to make an informed choice are as follows.

### Cost

In a study done in United Kingdom by Vidal-Alaball et al. (23), using oral vitamin B12 after diagnosis or switching from IM route could save resources in the medium and long term. The use of oral route results in significant reduction in manpower costs.

### Reduction of Scheduled Visits to Clinics

Many patients with vitamin B12 deficiency are elderly and have multiple co-morbidities. They often have multiple appointments to attend various clinics and may have frequent hospitalization episodes. The need to schedule vitamin B12 injections is an avoidable addition to the cost and complexity of their care.

### Adherence

In patients with non-compliance to oral medication, IM route may be a better option to ensure timely administration. On the other hand, oral replacement may improve adherence for patients who prefer oral medication to injections.

### Discomfort/Pain

Oral replacement will be useful in patients who are averse to injection. For elderly patients with sarcopenia, injections can painful and difficult to administer.

### Risk Associated with IM Injection

In patients, whereby IM injections are contraindicated because of coagulopathy or the use anti-coagulation/anti-platelet medication. Oral replacement is the best option.

## Limitations

1. The review was conducted only using PubMed and hand search.
2. Studies were mainly on patients with vitamin B12 deficiency with a subset of patients with pernicious anemia. Therefore, the actual sample size of pernicious anemia patients in each study may not reach statistical significance. We overcome this by triangulating our conclusions and recommendations based on the findings from multiple studies and the consistent oral vitamin B12 dosage of 1000 μg.
3. Many of the studies had small sample size and assessed only short-term outcomes. The long-term efficacy and side effects require further evaluation.
4. A few possible relevant articles were excluded due to language issues.

## Direction of Future Research

A multicenter randomized clinical trial is current in progress in Spain primary health-care setting called Project OB12 (24). This study aims to provide a more conclusive answer with a large sample size of 320 patients and longer follow-up period of 52 weeks. Further studies should include testing the efficacy of different dosages. It may be important to study the knowledge and practices of doctors/health-care workers with regard to oral replacement therapy with vitamin B12 for patients with pernicious anemia. Surveys on patients’ preferences for oral or IM replacement would be informative to guide clinical decision-making.

## Conclusion

Oral vitamin B12 replacement at 1000 μg daily is an adequate alternative to IM B12 injections. Close monitoring with clinical review and repeat vitamin B12 levels are required on a monthly basis to review symptoms and ensure normalization of B12 deficiency. Elevated serum Hcy and MMA levels should be included in future assessments of pernicious anemia and corrected with normal levels in patients with pernicious anemia.

## Author Contributions

Conceived and designed the study: CC, LL, and KL. Performed the study: CC and LL. Analyzed the data: CC and LL. Interpreted the results: CC and LL. Wrote the paper: CC, LL, and KL. Principal Investigator of this study and supervised this study: CC. Revised the paper critically and give final approval for publication: all authors.

## Conflict of Interest Statement

The authors declare that the research was conducted in the absence of any commercial or financial relationships that could be construed as a potential conflict of interest.

## Abbreviations

RCT, randomized controlled trial.
